# Overcoming degradation in spatial multiplexing systems with stochastic nonlinear impairments

**DOI:** 10.1038/s41598-018-35893-4

**Published:** 2018-12-03

**Authors:** Filipe M. Ferreira, Christian S. Costa, Stylianos Sygletos, Andrew D. Ellis

**Affiliations:** Aston Institute of Photonic Technologies, Aston University, Birmingham, B47ET United Kingdom

## Abstract

Single-mode optical fibres now underpin telecommunication systems and have allowed continuous increases in traffic volume and bandwidth demand whilst simultaneously reducing cost- and energy-per-bit over the last 40 years. However, it is now recognised that such systems are rapidly approaching the limits imposed by the nonlinear Kerr effect. To address this, recent research has been carried out into mitigating Kerr nonlinearities to increase the nonlinear threshold and into spatial multiplexing to offer additional spatial pathways. However, given the complexity associated with nonlinear transmission in spatial multiplexed systems subject to random inter-spatial-path nonlinearities it is widely believed that these technologies are mutually exclusive. By investigating the linear and nonlinear crosstalk in few-mode fibres based optical communications, we numerically demonstrate, for the first time, that even in the presence of significant random mixing of signals, substantial performance benefits are possible. To achieve this, the impact of linear mixing on the Kerr nonlinearities should be taken into account using different compensation strategies for different linear mixing regimes. For the optical communication systems studied, we demonstrate that the performance may be more than doubled with the appropriate selection of compensation method for fibre characteristics which match those presented in the literature.

## Introduction

The Kerr nonlinear limit has imposed an ever-growing capacity gap between the technologies generating/processing data and the technologies transporting it – namely, optical fibre communication systems. The first has consistently grown at 40% compound annual growth rate (CAGR)^1^, but the latter has slowed to 20% CAGR since late 1990s^2^. Such large scaling disparity is expected to lead to a full exhaustion of system capacity within the next 5 to 15 years. By 2024, optical networks are projected to require 1 Pb/s transmission capacity which with current technological limits can be expected to be met using 10 parallel line systems each carrying 100 Tb/s per fibre. A trend that would potentially increase the cost- and energy-per-bit by 10 times except for efficiency gains as ancillary functions overhead^3^ is reduced via sub-system integration. However, with the number of required spatial paths projected to double every 2-years and the current communications infrastructure accounting for 1–2% of global energy^4^ the current paradigm is exhausted. Thus, research effort must be directed towards the development of transformative means for achieving spatial parallelism that can ensure sublinear scaling of the total system cost and energy consumption. Otherwise, the dooming capacity exhaustion will lead to a dramatic increase of the bandwidth price and ultimately bring the information revolution to a halt.

**Figure 1:**
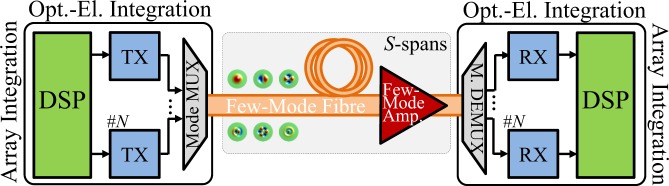
Basic system concept for a multi-span MDM system. MDM offers the possibility of high-density integration of the optical-electronic components and of efficient exploitation of shared DSP to simultaneously mitigate FMF specific impairments and array-specific impairments.

Mode-division multiplexing (MDM) over few-mode fibres (FMFs) holds one the greatest potential to deliver future cost- and energy-effective high-capacity systems with spatial parallelism^5,6^. Figure 1 shows the basic system concept of a multi-span MDM-FMF system, composed by integrated arrays of *M* transmitter and *M* receiver units, mode multiplexers/de-multiplexers (e.g. photonic lanterns^7^), and multimode amplifiers^8^. The information is carried over a set of orthogonal spatial modes overlapping on a single fibre core. Compared to alternative technologies, such as uncoupled multi-core fibres or single-mode fibre (SMF) bundles^5^, MDM-FMF systems offer a number of advantages, such as lower nonlinear coefficients; higher pump efficiency for their optical amplifiers (similar to core pumped SMF)^9^; and higher spatial-density level of optical integration for transponders^10^, amplifiers, and add-drop multiplexers (multiple spatial modes can be routed together^11^). Nevertheless, coupled-core multi-core fibres (CC-MCFs) offer similar potential to that of FMFs when designed to have similar spatial mode densities^12–14^. Finally, the techniques presented in this paper apply to all SDM fibre types, including CC-MCFs.

**Figure 2:**
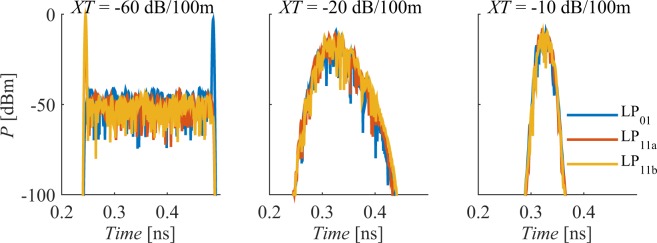
Channel impulse response of a 50 km long three-mode fibre with a DMD of 5 ps/km, given the simultaneous launch of three Gaussian pulses (2.5 ps FWHM), one per mode, with XT equal to: (**a**) −60 dB/100 m, (**b**) −20 dB/100 m, and (**c**) −10 dB/100 m.

Despite the potential of MDM-FMF technology, commercialisation and deployment is not imminent yet because whilst the fibres are now filling up, there are spare fibres in the network, giving a few years grace before an expensive installation of new fibre becomes necessary. By the end of that period, considering the 45% CAGR of CMOS processing capabilities^10^, it is likely that the DSP ASICs will be able to handle 10 Tb/s worth of traffic by directly coupling to arrays of 10 (or more) parallel integrated high-speed (~Tb/s) optical and opto-electronic components (such as modulators or coherent receivers). Processing multiple tributaries simultaneously would allow to compensate^15,16^ the combined effects of mode-mixing and walk-off between the different parallel fibre paths shown in Fig. 2. Presently, differential mode delay (DMD) and linear mode-crosstalk (XT) have been successfully mitigated with offline multi-input multi-output (MIMO) based DSP techniques^16,17^ and DMD compensation maps after transmission over thousands of kilometres^18,19^. However, whilst SDM systems have seen a significant growth of their bit-rate distance product (e.g. 115 Petabit/s∙km using a 6-mode 19-core fibre^20^ and 166 Petabit/s∙km using a 3-mode single-core fibre^21^), they have yet to match the records established using SMFs (e.g. 535 Petabit/s∙km in^22^). This is due to the increased mode dependent loss of the prototype components^19,23^ used in a research lab environment, see Fig. 1, and due to the intermodal nonlinear interactions^18,24,25^ that occur in the transmission medium. However, given the engineering development of prototype components^26–29^ (for example, monolithic mode-selective few-mode multicore fibre multiplexers with insertion loss <2 dB and mode dependent loss <0.5 dB have been recently demonstrated over the C + L band with identified margin for improvement^29^), the impact of the former will become smaller and the latter will therefore become dominant. The intermodal nonlinear distortion is most relevant when the system is loaded with a sufficiently high number of channels such that the total system bandwidth allows exact cancelling of chromatic dispersion and DMD walk-offs for all pairs of modes. Thus, in order to realise the full potential of FMF system by simultaneously increasing the information spatial density (Gbit/s/cm^2^) and maintaining the system reach it is of paramount importance to address the nonlinear penalties.

**Figure 3:**
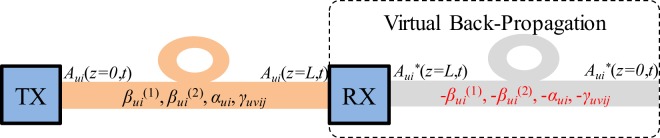
Digital-back propagation basic concept.

Digital-back propagation (DBP) is a nonlinear mitigation method originally proposed for SMFs^30^ that compensates for the deterministic linear and nonlinear fibre impairments by numerically back-propagating the received optical field with inverted channel parameters, using the split-step Fourier method (SSFM) to solve the nonlinear propagation equation, as shown in Fig. 3. However, effectiveness of this technique is reduced in the presence of random processes such as the group delay spread induced by DMD and XT, like polarisation mode dispersion (PMD) in SMFs^31–33^. A brute force approach, following recent attempts to mitigate PMD^31,34^, would be to estimate in the digital domain the slowly varying differential delays and the rapidly varying random mixing on relatively short length scales. However, this would increase the complexity, due to the short step size required, and would accumulate an increasing numerical error due to the large number of estimates. In FMFs with large DMD between non-degenerate modes this problem can become much more pronounced depending on the strength of the random linear mode coupling since in its absence the optical signal evolution is fully predictable and DBP could compensate for the nonlinear distortion perfectly (ignoring transceiver noise). In this way, one can expect the amount of DMD tolerable by DBP systems to be much higher for FMFs operating in the weak linear coupling regime than for FMFs operating in the strong linear coupling regime (depending on the DBP implementation).

In this paper, we introduce new simplified DBP methods for the different operational regimes of MDM-FMF systems as determined by the strength of DMD and XT. To illustrate their benefit, we consider the transmission of wavelength-division multiplexed (WDM) multi-level quadrature amplitude modulated (QAM) signals. Some of the proposed methods have been considered in a preliminary study^35^ under simplified conditions that include the absence of amplified spontaneous noise, and constant amplitude signals.

**Figure 4:**
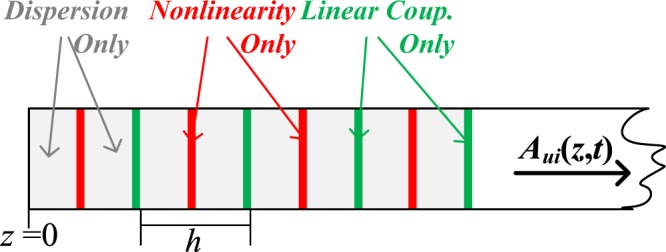
Schematic illustration of the symmetric SSFM used for numerical simulations.

In this paper, we use an extension of the single-mode split-step Fourier method (SSFM)^36^ to numerically solve the generalized nonlinear Schrödinger equation (GNLSE) that governs multimode propagation over FMFs^37^ (including all linear and nonlinear fibre effects), provided that the split-step size is compatible with the additional requirements, see Methods. To achieve higher accuracy in the modelling of forward propagation over a real fibre, we consider a symmetric implementation of the SSFM in which the effect of nonlinearity is included in the middle of the segment rather than at the segment boundary^38^, see Fig. 4. Finally, for backward propagation over the virtual fibre (with inverted parameters), GNLSE can be simplified depending on the strength of the linear mode coupling, see Methods, reducing the computational requirements.

In the presence of extreme linear mode coupling regimes, it has been shown that some or all the linear mode coupling terms in GNLSE can be assumed to vary rapidly and seemingly randomly on a length scale that is expected to be short compared to the effective lengths associated with chromatic dispersion and the various manifestations of nonlinearity. Thus, like in single-mode fibres and the well-known Manakov-PMD equations^39,40^, one can average the propagation equation itself over all spatial modes. New Manakov-like equations were derived for FMFs^41,42^ with nonlinear coefficients averaged for the two extreme coupling regimes. In the weak coupling (WC) regime^41^, only the averaging over birefringence fluctuations must be considered, reducing the intramodal degeneracy factor to 8/9 and the intermodal degeneracy factor to 4/3, see Methods. In the strong coupling (SC) regime the averaging includes all propagation modes^42^, see Methods.

In the intermediate linear coupling regime, the linear mode coupling introduced by GNLSE needs to be explicitly considered in the SSFM. It has been shown that the semi-analytical solution of this term is possible by discretising the fibre imperfections responsible for linear coupling as fibre sections with a random displacement of the core centre position^37^. In this way, the authors proposed a multi-section model where the coupling strength is set using a given radial displacement and a uniformly distributed azimuthal displacement for each section. More importantly, the proposed model proved to be accurate against analytical predictions for the statistics of group-delays in FMF links. Finally, the linear coupling step in the SSFM used in this paper is implemented following^43^.

To compensate for the GD spread imposed by the interplay of XT and DMD, see Fig. 2, multi-input multi-output (MIMO) digital signal processing (DSP) is used with training-symbol-based frequency-domain equalisers offering the lowest complexity^44^. However, as the effective transmission rate is reduced by the additional training symbols overhead, low DMD links must be used. It has been shown that a DMD lower than 12 ps/km is required for 2000 km MDM transmission at 100 Gb/s in order to restrict the training sequences overhead to 10%^44,45^. In this way, significant effort has been directed to the optimisation of FMFs, with refractive-index profiles typically composed of a graded-index core (for DMD reduction) and a cladding trench (for macro-bend loss reduction via field confinement). Ferreira *et al*. in^14^ have optimised such profile to guide six linearly polarized (LP) modes (LP_01_, LP_02_, LP_11a_, LP_11b_, LP_21a_ and LP_21b_) with low *DMD* (12 ps/km for GD unmanaged long-haul transmission^44^) and macro-bend losses as low as in SMFs^46^. Here, we use the same optimum profile. Table 1 shows the optimum profile linear characteristics at 1550 nm, the *DMD* defined as max(*GD*)-min(*GD*) is 5.19 ps/km. Table 2 shows the uncoupled nonlinear coefficients whilst the uncoupled degeneracy factors are found in the GNLSE, see Methods. In this paper, when considering different *DMD* values, we simply scale the GD vector in Table 1 instead of reoptimizing the fibre profile (as in^14^) to avoid fluctuations of the other fibre characteristics, such that a direct performance assessment of the proposed DBP methods can be accomplished.

**Table 1:** Fibre Linear Characteristics at 1550 nm.

	LP01	LP02	LP11a	LP11b	LP21a	LP21b
GD [ps/km]	−0.29	−2.93	−0.66	−0.66	2.27	2.27
*D* [ps/(nm.km)]	22.18	21.55	22.15	22.15	21.84	21.84
*S* [fs/(nm^2^.km)]	66.45	61.46	66.15	66.15	63.68	63.68
α [dB/km]	0.20	0.20	0.20	0.20	0.20	0.20

**Table 2:** Nonlinear Coeffs (*γ*_*uv*_) at 1550 nm [W^−1^/km].

*u v*	LP01	LP02	LP11a	LP11b	LP21a	LP21b
LP01	0.73	0.36	0.36	0.36	0.18	0.18
LP02	0.36	0.36	0.18	0.18	0.18	0.18
LP11a	0.36	0.18	0.55	0.18	0.27	0.27
LP11b	0.36	0.18	0.18	0.55	0.27	0.27
LP21a	0.18	0.18	0.27	0.27	0.41	0.14
LP21b	0.18	0.18	0.27	0.27	0.14	0.41

Due to current estimations of the fibre manufacturing limitations^14^ it is challenging to achieve *DMD* <12 ps/km for more than 3 LP modes, therefore GD managed spans are often used to minimise the total GD spread by cascading fibres with opposite sign DMD^19,47,48^. Here we define a GD managed span of length *L* as one comprising *S* segments, each itself is composed of two fibres of length *L*/*S*/2 with the same characteristics but opposite sign GD (in practice for fibres with more than 2 non-degenerate LP modes, more than two different fibres are needed^19,47,48^ since exact opposite sign GD is hard to achieve). The GD spread at the end of a managed span is only zero in the absence of linear mode coupling, otherwise there is a residual GD spread. To minimise mode coupling impact, the GD compensation length must be smaller than the correlation length set by the coupling^49^, which might not be practical when correlation length is on the order of a km. In this paper, when considering *DMD* >10 ps/km, we will consider one compensation segment per span.

**Figure 5:**
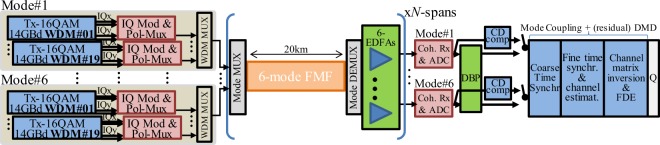
Simulation setup for 6 LP modes each with 2 orthogonal polarisations, 19 WDM channels, including: optical transmitter, mode multiplexer (MUX) and demultiplexer (DEMUX), FMF, coherent receiver, and DSP blocks.

Along this study we consider a WDM-MDM system with 6 linearly polarized (LP) modes each with two orthogonal polarisations. The simulation setup is shown in Fig. 5, where 12.8 Tbit/s are transmitted over 19 WDM channels (in each spatial mode) modulated with 14 Gbaud polarisation-multiplexed 16QAM. Together with the information data, a preamble was transmitted consisting of constant amplitude zero autocorrelation (CAZAC) sequences for time synchronization and channel estimation. Root raised cosine filters with a roll-off factor of 0.001 were used for pulse shaping. For the simulations we considered 2^16^ symbols per polarisation mode, with a 2^11^ symbols CAZAC preamble. The in-phase and quadrature components of each signal drove the optical field of an ideal laser through an optical IQ modulator, and the optical signals were fed into the FMF link (non-GD-managed and GD-managed links are considered). Fibre attenuation was fully compensated using an array of 6 erbium doped fibre amplifiers^19^, with noise figures of 3 dB and negligible mode dependent loss since the aim of this paper is to assess the isolated impact of the FMF mode coupling and mode delay on DBP performance. The optical signals were coupled in and out of the FMF using a mode multiplexer (MUX) and a mode demultiplexer (DEMUX), respectively, assumed ideal since their transfer function can be fully compensated through DSP given prior characterization (as in conventional transceivers^50^). After homodyne detection, the baseband electrical signals were sampled at 56 GS/s, yielding 12 digital signals at 2 samples/symbol. DBP was then implemented by launching the coherently received signals into a virtual fibre with characteristics of opposite-sign values of those in the transmission channel, except that no mode coupling was considered. Back-propagation was implemented using the modified SSFM proposed with a fixed step size and considering the nonlinear degeneracy factors derived for WC and SC, Eqs (3) and (4), respectively. As a reference, for linear compensation, the coherently received signals were compensated for chromatic dispersion in the frequency domain using the parameter values of Table 1. In all cases, mode coupling and (residual) DMD were subsequently compensated for using training-symbol-based channel estimation and equalization, as shown in Fig. 5. Coarse time synchronization was performed using the Schmidl & Cox autocorrelation metric. Subsequently, fine-time synchronization and channel impulse response (CIR) estimation were performed by cross-correlating with the training CAZAC sequences. The 12 × 12 CIR estimations were converted into the frequency domain. The MIMO frequency domain equalizer was calculated by inverting the channel matrix, and, finally, the Q-factor for each received signal was estimated using the mean and standard deviation of the received symbols^51^.

## Results

DBP performance was studied on an optical super-channel consisting of 19 channels (in each of the 6 LP modes), 14 Gbaud polarisation-multiplexed 16QAM (with a frequency spacing of 14.1 GHz), corresponding to a total line-rate of 12.8 Tb/s (a net-data-rate of 9.8 Tb/s given 20% FEC and 3.1% training sequences overheads), over 12 spans of 20 km (the required energy per bit is minimised for span lengths going from 35 km at 6 bits/s/Hz to 20 km at 12 bits/s/Hz^52^). With a total WDM bandwidth of 268 GHz, the centre WDM channels will experience intermodal nonlinear interactions for all possible combinations of pairs of modes when considering an overall *DMD* of up to 21.4 ps/km (to allow exact cancelling of chromatic dispersion and DMD walk-offs within the system bandwidth). Conversely, to make all possible intermodal FWM negligible, the overall *DMD* as to be increased to be higher than 300 ps/km such that the *DMD* between LP_01_ and LP_11a/b_ (this is, the mode pair with closest group delay in Table 1) exceeds 21.4 ps/km. The study considered DBP implemented using the WC-Manakov Eq. (3)^41^ (WC-DBP), the SC-Manakov Eq. (3)^42^ (SC-DBP), or just the intra-modal nonlinear coefficients in WC-Manakov Eq. (3) (Intra-DBP), and considered as figure of merit the *Q-factor* of the centre channels averaged over the 12 polarisation modes. The fibre mode coupling strength (*XT*) was swept within a broad range of values, i.e. −60 dB/100 m to 0 dB/100 m, covering the weak, intermediate and strong coupling regimes, thus covering all observed coupling values presented in the literature^53–58^. For forward propagation, the step size was selected by bounding the local error^38^, a method found to be more computationally efficient at high accuracy than other common methods such as nonlinear phase rotation. The local error was bound to be lower than 10^−5^ as smaller values led to negligible performance change. Conversely, the maximum step size is kept much smaller than the dispersion length, the walk-off length, and the correlation length, as explained in Methods. For backward propagation, a constant step size is used, its value is determined in the following. In all cases, backpropagation considered the total number of channels being transmitted since multi-channel DBP was found to be required to achieve effective nonlinearity mitigation for SMF systems^59^ and this paper aims at exploring the full potential of DBP in SDM systems.

**Figure 6:**
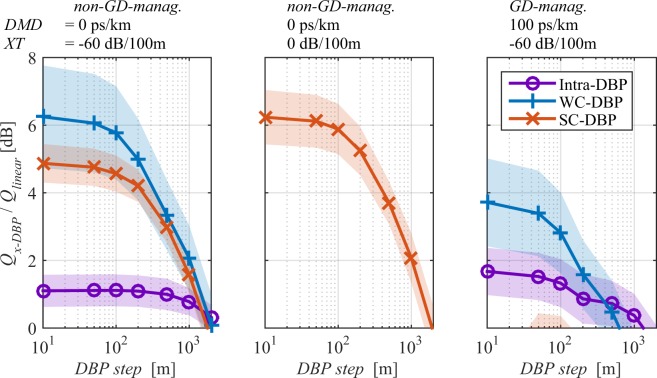
*Q-factor* gain over linear equaliser for WC- and SC-DBP as a function of the DBP step after 240 km for 0 dBm/ch and for different combinations of *XT*, *DMD* and *GD* management segments. Lines shadow accounts for 3 times the standard deviation for 40 repetitions.

Figure 6 shows the *Q-factor* gain over linear equalisation for WC-, SC-, and Intra-DBP as a function of the DBP step size after 240 km transmission with a launch power of 0 dBm/ch, for two DMD free fibre links, (a) one with very low *XT* and (b) one with very high *XT*, and for (c) one high *DMD* fibre link GD-managed (one segment). These cases are representative of the broader range of *DMD* and *XT* values considered in the following. In all cases, results suggest that the simulation had converged by 100 m (similar results to 10 m). Thus, from this point, the step was kept at 100 m as this paper aims at exploring the full potential of digital nonlinear mitigation.

**Figure 7:**
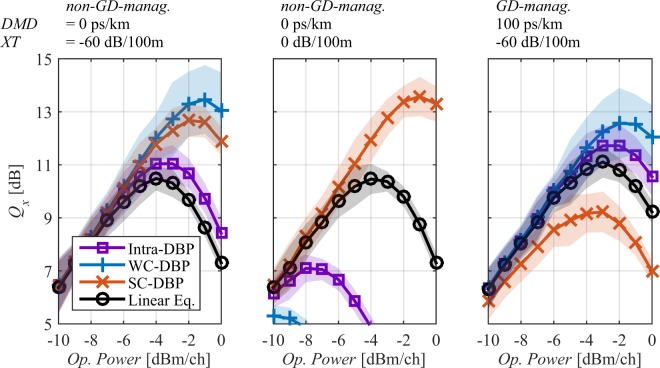
*Q-factor* as a function of the power per channel after 240 km for different combinations of *XT*, *DMD* and *GD* management segments. Lines shadow accounts for 3 times the standard deviation for 40 repetitions.

Figure 7 shows the Q-factor as a function of the power per channel (*P*_*ch*_) after 240 km for two DMD free fibre links, (a) one with very low *XT* and (b) one with very high *XT*, and for (c) one high *DMD* fibre link GD-managed (one segment). From Fig. 7(a,b), it can be seen that WC- and SC-DBP provide substantial *Q-factor* improvement in particular for low *XT* and high *XT* respectively, as Manakov approximations are applicable. And, that Intra-DBP provides performance improvement only for low *XT* but of smaller order as intermodal nonlinear interactions (strong for low *DMD*) are not accounted while DBP. Moreover, one can observe that WC-DBP only provides gain for transmission over the weakly coupled fibre, while SC-DBP provides gain for both fibres. WC-DBP is particularly penalizing for high *XT* values as the nonlinear coefficients in Eq. (2) are larger than the actual channel coefficients leading to large overcompensation. SC-DBP provides gain even for low *XT* as the nonlinear coefficients in Eq. (3) are smaller than the actual channel coefficients leading to undercompensation. From Fig. 7(c), it can be concluded that when using GD management with high *DMD* and low *XT* (GD management with high *XT* is not effective^43^), WC-DBP allows to achieve maximum *Q-factors* of the same order of those for low *DMD* non-GD-managed spans (Fig. 7(a)). Also in Fig. 7(c), as intermodal FWM efficiency is reduced for high *DMD* (and a given system bandwidth, as explained previously), Intra-DBP produces similar gains to those of WC-DBP. Finally, in Fig. 7(a,c), the large errors bars for WC-DBP are a consequence of having extremely low strength mode mixing (−60 dB/100 m) providing a few additional pathways to intermodal four-wave mixing phase matching without introducing sufficiently fast random rotations of the field polarisation state along the fibre length which would have led to an averaging of the efficiency of the overall nonlinear process. Note that in practice the lower bound of the *Q-factor* variation would be considered in the dimensioning of the system. Note that in practice the lower bound of the *Q-factor* variation would be considered in the dimensioning of the system.

**Figure 8:**
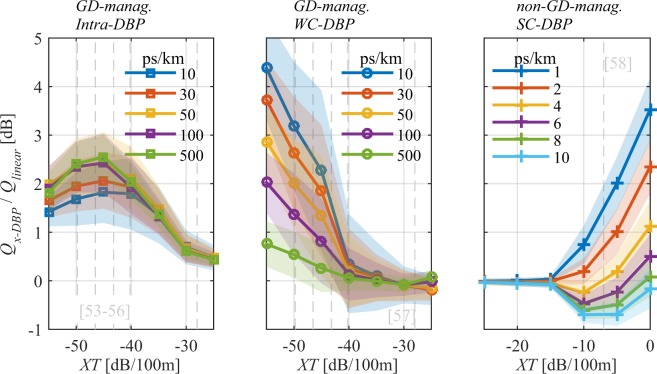
*Q-factor* gain over linear equaliser as a function of *XT* after 240 km, 0 dBm/ch and different *DMD* values, with: (a) Intra-DBP, (b) WC-DBP and (c) SC-DBP. Lines shadow accounts for 3 times the standard deviation for 100 repetitions.

Figure 8 shows the *Q*-factor improvement over linear equalisation as a function of *XT* after 240 km with different values of *DMD* and launch power of 0 dBm/ch for: (a) Intra-, (b) WC- and (c) SC-DBP. First, it can be seen that WC- and SC-DBP can provide significant compensation (above 1 dB) in the regimes where their Manakov equations are valid (for *XT* < −40 dB/100 m and *XT* > −15 dB/100 m, respectively). But, also that Intra-DBP provides a performance improvement in many cases higher than that of WC-DBP. Intra-DBP performs particularly well for sufficiently high *DMD* such that intermodal nonlinear distortion is not so dominant and for a range of low *XT* in which sufficient coupling events randomise a sufficient share of the intermodal nonlinear distortion. Thus, for sufficiently low *XT*, Intra-DBP gain rolls-off as can be seen in Fig. 8(a). In this way, for the WC-regime, and for fibres with −55 < *XT* [dB/100 m] < −35 and *DMD* > 30 ps/km Intra-DBP provides the highest improvement between 1 and 3 dB, and for fibres with *XT* < −40 dB/100 m and *DMD* < 30 ps/km WC-DBP provides an improvement between 1 and 4 dB. These *XT* and *DMD* ranges cover many the fibres presented in literature^53–58^. For the SC-regime, SC-DBP provides significant compensation for small *DMD* values (≤4 ps/km) and *XT* ≥ −10 dB/100 m, a regime that can be achieved using for example a fibre similar to the one in^58^. For intermediate *XT* values (−30 < *XT* [dB/100 m] < −15) none of the DBP approaches work even for negligible *DMD* values. This is because for significant transmission distances (240 km, in this case) linear mode coupling leads to evolutions of the nonlinear operator that differ significantly from that of the uncoupled operators in the Manakov approximation. Outside the operational regime identified for WC- and SC-DBP, the evolution of the GD operator is no longer well approximated using the uncoupled GD coefficients as in Table 1, thus the nonlinear distortion is either overcompensated or undercompensated when using the coefficients in Table 2 or their direct average^41,42^.

## Discussion

Even for the most complex MDM systems significant performance improvement is possible using DBP provided that appropriate approximations for the effect of the stochastic nature of the linear crosstalk are taken into account. For example, fibres optimised primarily for low *XT* (and with intermediate-to-high *DMD*), including trench-assisted graded-index fibres^19,53^ or multiple-step index fibres^54,60^, allow a significant DBP gain if the crosstalk is neglected. However, this signal processing approach gives no gain for high *XT* (and low *DMD*) fibres such as coupled-core fibres^18,58^. However, for such high *XT* fibres, if the instantaneous crosstalk is averaged, the so called generalised Manakov approach, high performance gains are again possible. Whilst a small range of possible fibre parameters exist where the approximate models considered here failed to provide significant gain, and compensation would require continuous estimation of the random linear coupling, significant performance gains were possible for all possible XT and DMD regimes in which real fibres operate.

To extend the operational range of Manakov-DBP, active tracking of the GD operator is needed, as observed for SMF systems impacted by polarisation mode dispersion (PMD)^31,34^. The DBP algorithm proposed in^31^, takes into account PMD by simply considering GD accumulating in staircase fashion, span-by-span, in such a way that the total PMD accumulated in the forward propagation is reversed (total PMD is available at conventional linear channel equalizers). In^34^, instead of considering GD accumulating over the same principal states of polarisation in the backward propagation, a blind optimization of each section Jones matrix improved performance by reducing the DBP gain variability at the expense of additional complexity. Future research to develop methods similar to^34^ applicable to MDM systems are expected to deliver significant additional gains (>1 dB) over a broad range of XT and DMD regimes.

## Methods

### Generalized Nonlinear Schrödinger Equation

The generalized nonlinear Schrödinger equation (GNLSE) for FMFs can be written as^37^:

1 $$\begin{array}{l}{\partial }_{z}{A}_{ui}+\mathop{\mathop{[{\rm{j}}{\beta }_{ui}^{(0)}+{\beta }_{ui}^{(1)}{\partial }_{t}-\frac{{\rm{j}}{\beta }_{ui}^{(2)}}{2}{\partial }_{t}^{2}+\ldots +\frac{{\alpha }_{ui}}{2}]}\limits^{\frown {}}}\limits^{=\hat{D}}{A}_{ui}\\ \begin{array}{rcl} & = & -\,{\rm{j}}\mathop{\mathop{[{\gamma }_{uuii}{|{A}_{ui}|}^{2}+2{\gamma }_{uvii}\sum _{v\ne u}{|{A}_{vi}|}^{2}+\frac{2}{3}{\gamma }_{uvij}\sum _{\nu }{|{A}_{vj}|}^{2}]}\limits^{\frown {}}}\limits^{=\hat{N}}{A}_{ui}\\  &  & -\,{\rm{j}}\mathop{\mathop{\sum _{vk}{C}_{uvik}{A}_{vk}{e}^{{\rm{j}}({\beta }_{ui}^{(0)}-{\beta }_{vk}^{(0)})z}}\limits^{\frown {}}}\limits^{=\hat{C}},\end{array}\end{array}$$

where *i* and *j* are the orthogonal states of polarisation of each mode *u* and *v*, with *u*, *v* = (1, …, *N*) for *N* linearly polarised modes each two orthogonal polarisations. In this way, (1) is a set of 2*N* coupled equations, one for each polarisation mode *ui*. *A*_*ui*_(*z*,*t*), *β*_*ui*_^(1)^, *β*_*ui*_^(2)^ and *α*_*ui*_ are the slowly varying field envelope, group delay, group delay dispersion and attenuation, respectively. *γ*_*uvij*_ is the nonlinear coefficient between *ui* and *vj*, which depends on the nonlinear refractive index *n*_2_ of the silica, approximately 2.6 × 10^−26^ m^2^/W, and on the intermodal effective area, and is given by:

2 $${\gamma }_{uvij}={n}_{2}\frac{{\omega }_{0}}{c}\frac{{\iint }_{-\infty }^{+\infty }{|{E}_{ui}|}^{2}{|{E}_{vj}|}^{2}{\rm{d}}x{\rm{d}}y}{({\iint }_{-\infty }^{+\infty }{|{E}_{ui}|}^{2}{\rm{d}}x{\rm{d}}y)({\iint }_{-\infty }^{+\infty }{|{E}_{vj}|}^{2}{\rm{d}}x{\rm{d}}y)}$$

where *E*_*ui*_(*x*, *y*) is the mode field transverse distribution for the *i* polarisation of mode *u*.

In Eq. (1), $\hat{D}$ is the differential operator that accounts for dispersion and attenuation, and $\hat{N}$ is the nonlinear operator that accounts for all the intramodal and intermodal nonlinear effects^37^. The last term on the right-hand side accounts for the linear mode coupling arising from fibre structure imperfections, where *C*_*uvij*_ are the coupling coefficients as derived in^37^.

For the extreme coupling regimes, the nonlinear coefficients and degeneracy factors in (1) can be assumed being averaged by the linear coupling^41,42^, obtaining the so-called few-mode Manakov equations. In the weak coupling (WC) regime^41^, only the averaging over birefringence fluctuations must be considered, reducing the *intramodal* degeneracy factor to 8/9 and the intermodal degeneracy factor to 4/3. Thus, the nonlinear operator in (1) becomes:

3 $$\hat{N}=-\,{\rm{j}}[\frac{8}{9}\sum _{k=\{i,j\}}{\gamma }_{uuik}|{A}_{uk}{|}^{2}+\frac{4}{3}\sum _{\begin{array}{c}\nu \ne u\\ k=\{i,j\}\end{array}}{\gamma }_{u\nu ik}|{A}_{\nu k}{|}^{2}].$$

In the strong coupling (SC) regime, the averaging includes all propagation modes. For *N*-modes with two polarisations, the nonlinear operator in (1) becomes^42^:

4 $$\hat{N}=-{\rm{j}}\sum _{\begin{array}{c}\nu \\ k=\{i,j\}\end{array}}\kappa {|{A}_{\nu k}|}^{2},\,\kappa =\frac{4}{3}\frac{2N}{2N+1}(\frac{1}{{N}^{2}}\sum _{\begin{array}{c}u,\nu \\ k,l=\{i,j\}\end{array}}{\gamma }_{u\nu kl})$$

### Few-Mode Symmetric Split-Step Fourier Method

The single-mode split-step Fourier method (SSFM) obtains an approximate solution of the Schrödinger equation by assuming that over a small distance *h* the dispersive and nonlinear effects act independently. For FMFs, we extend such an approach by assuming that the mode coupling also acts independently. Such approximations require *h* to be much shorter than the dispersion length *T*_0_^2^/|*β*_*u*_^(2)^| and the walk-off length *T*_0_/|*β*_*u*_^(1)^ − *β*_*v*_^(1)^| where *T*_0_ is the pulse width, and shorter than the correlation length *L*_*c*_ defined^24^ for *XT*(*L*_*c*_) = [*e*^2^ − 1]/[*e*^2^ + 1].

Figure 1 presents a schematic illustration of the few-mode symmetric SSFM proposed. In a symmetric SSFM, the effect of nonlinearity is included in the middle of the segment rather than at the segment boundary^38^, providing higher accuracy. Finally, the step-size was selected by bounding the local error^38^, more computationally efficient at high accuracy than the other methods, e.g. nonlinear phase rotation.
